# Supplementary material for: OpenMM 7: Rapid development of high performance algorithms for molecular dynamics
Source: PLoS Comput Biol. 2017 Jul 26;13(7):e1005659. doi: 10.1371/journal.pcbi.1005659 (PMC5549999; doi:10.1371/journal.pcbi.1005659)
Supplement: S1 Source Code — (ZIP) [file pcbi.1005659.s002.zip › OpenMM-7.0.1-Source/docs/api-python/generated/simtk.openmm.openmm.LocalCoordinatesSite.html]

LocalCoordinatesSite — OpenMM 7.0.1.dev-5e86c4f documentation


# LocalCoordinatesSite¶

*class* `simtk.openmm.openmm.``LocalCoordinatesSite`(*\*args*)¶
:   This is a VirtualSite that uses the locations of three other particles to compute a local coordinate system, then places the virtual site at a fixed location in that coordinate system. The origin of the coordinate system and the directions of its x and y axes are each specified as a weighted sum of the locations of the three particles:

    origin = w<sub>1</sub>r<sub>1</sub> + w<sub>2</sub>r<sub>2</sub> + w<sub>3</sub>r<sub>3</sub>

    xdir = w<sub>1</sub>r<sub>1</sub> + w<sub>2</sub>r<sub>2</sub> + w<sub>3</sub>r<sub>3</sub>

    ydir = w<sub>1</sub>r<sub>1</sub> + w<sub>2</sub>r<sub>2</sub> + w<sub>3</sub>r<sub>3</sub>

    For the origin, the three weights must add to one. For example if (w<sub>1</sub>, w<sub>2</sub>, w<sub>3</sub>) = (1.0, 0.0, 0.0), the origin of the local coordinate system is at the location of particle 1. For xdir and ydir, the weights must add to zero. For excample, if (w<sub>1</sub>, w<sub>2</sub>, w<sub>3</sub>) = (-1.0, 0.5, 0.5), the x axis points from particle 1 toward the midpoint between particles 2 and 3.

    The z direction is computed as zdir = xdir x ydir. To ensure the axes are all orthogonal, ydir is then recomputed as ydir = zdir x xdir. All three axis vectors are then normalized, and the virtual site location is set to

    origin + x\*xdir + y\*ydir + z\*zdir

    `__init__`(*self*, *particle1*, *particle2*, *particle3*, *originWeights*, *xWeights*, *yWeights*, *localPosition*) → LocalCoordinatesSite¶
    :   \_\_init\_\_(self, other) -> LocalCoordinatesSite

        Create a new LocalCoordinatesSite virtual site.

        |  |  |
        | --- | --- |
        | Parameters: | - **particle1** (*int*) – the index of the first particle - **particle2** (*int*) – the index of the second particle - **particle3** (*int*) – the index of the third particle - **originWeights** (*Vec3*) – the weight factors for the three particles when computing the origin location - **xWeights** (*Vec3*) – the weight factors for the three particles when computing xdir - **yWeights** (*Vec3*) – the weight factors for the three particles when computing ydir - **localPosition** (*Vec3*) – the position of the virtual site in the local coordinate system |

    Methods

    |  |  |
    | --- | --- |
    | `__init__`((self, particle1, particle2, ...) | \_\_init\_\_(self, other) -> LocalCoordinatesSite |
    | `getLocalPosition`((self) -> Vec3 const &) | Get the position of the virtual site in the local coordinate system. |
    | `getNumParticles`((self) -> int) | Get the number of particles this virtual site depends on. |
    | `getOriginWeights`((self) -> Vec3 const &) | Get the weight factors for the three particles when computing the origin location. |
    | `getParticle`((self, particle) -> int) | Get the index of a particle this virtual site depends on. |
    | `getXWeights`((self) -> Vec3 const &) | Get the weight factors for the three particles when computing xdir. |
    | `getYWeights`((self) -> Vec3 const &) | Get the weight factors for the three particles when computing ydir. |

    `getOriginWeights`(*self*) → Vec3 const &¶
    :   Get the weight factors for the three particles when computing the origin location.

    `getXWeights`(*self*) → Vec3 const &¶
    :   Get the weight factors for the three particles when computing xdir.

    `getYWeights`(*self*) → Vec3 const &¶
    :   Get the weight factors for the three particles when computing ydir.

    `getLocalPosition`(*self*) → Vec3 const &¶
    :   Get the position of the virtual site in the local coordinate system.

    `__delattr__`¶
    :   x.\_\_delattr\_\_(‘name’) <==> del x.name

    `__format__`()¶
    :   default object formatter

    `__getattribute__`¶
    :   x.\_\_getattribute\_\_(‘name’) <==> x.name

    `__hash__`¶

    `__reduce__`()¶
    :   helper for pickle

    `__reduce_ex__`()¶
    :   helper for pickle

    `__sizeof__`() → int¶
    :   size of object in memory, in bytes

    `__str__`¶

    `getNumParticles`(*self*) → int¶
    :   Get the number of particles this virtual site depends on.

    `getParticle`(*self*, *particle*) → int¶
    :   Get the index of a particle this virtual site depends on.

        |  |  |
        | --- | --- |
        | Parameters: | **particle** (*int*) – the particle to get (between 0 and getNumParticles()) |
        | Returns: | the index of the particle in the System |
        | Return type: | int |

High performance molecular simulation on GPUs


### Navigation

- Application Layer
- Library Layer
  - Core Objects
  - Forces
  - Integrators
  - Extras

©2015, Stanford University and the Authors.
|
Powered by Sphinx 1.3.1
& Alabaster 0.7.7
|
Page source
